# On the relationship between various anticoagulants and robot-assisted radical prostatectomy: a single-surgeon serial analysis

**DOI:** 10.1007/s11701-024-01933-7

**Published:** 2024-04-13

**Authors:** Mahmoud Farzat, Florian M. Wagenlehner

**Affiliations:** 1 Department of Urology, Pediatric Urology and Andrology, Justus-Liebig University of Giessen, Wichernstraße 40, 57074 Siegen, Germany; 2 Department of Urology and Robotic Urology, Diakonie Klinikum, Siegen, Germany

**Keywords:** Anticoagulation, Prostate cancer, RARP, DAPT, Aspirin, NOAC, VKA

## Abstract

Prostate cancer patients often have other health conditions and take anticoagulants. It was believed that surgery under anticoagulants could worsen surgical results. This study aims to explore the safety of robot-assisted prostatectomy in anticoagulated patients, without any exclusion criteria. The study included 500 patients who underwent RARP by a single surgeon between April 2019 and August 2022. Patients were divided into two groups: Group 1, consisting of 376 men (75.2%), did not receive any anticoagulation, while Group 2, with 124 patients (24.8%), received different forms of anticoagulation. Then, the anticoagulation group was divided into 4 subgroups according to their definite anticoagulation: the aspirin 15.6%, new oral anticoagulants (NOAC) 5.4%, Vitamin K antagonist (VKA) 2%, and dual-antiplatelet therapy (DAPT) 1.8% subgroup. Postoperative complications and readmission rates were compared between the two study groups and subgroups. Patients in the combined group 2 were older and they also carried more comorbidities compared to men in group 1 (*p* = 0.03, *p* = 0.001).The study groups had similar oncological results, with 40.4% of patients having locally advanced cancers. Catheter days were longer in the anticoagulation group (4.5 vs 4 days, *p* = 0.001). No significant differences were observed between study groups for overall, minor, and major complications (*p* = 0.160, 0.100, and 0.915, respectively). In addition, readmissions were low (5.6%) and similar between the study groups (*p* = 0.635). Under cautious management, RARP under diverse anticoagulation regimes is safe and has comparable results to men with no medications. Further prospective studies must be conducted to confirm our findings.

## Introduction

Globally prostate cancer (PCa) is the most common cancer in men [1]. A large proportion of prostate cancer patients have comorbidities such as cardiovascular disease and coagulation disorders. Those conditions and consequently their therapy present a challenge for PCa-directed therapies whether systemic like antihormonal therapy or local like radical prostatectomy [2]. Other local therapy options like percutaneous radiation in combination with simultaneous androgen-deprivation therapy should also be critically recommended due to the cardiovascular side effects of hormonal deprivation [3]. Krane et al. [4] found that bridging with low molecular weight heparin (LMWH) in patients under Vitamin K antagonist (VKa) therapy and undergoing robot-assisted radical prostatectomy (RARP) increases bleeding and necessitates more transfusions (22 vs. 2%) [4]. Ning et al. [5] approached the subject differently in their meta-analysis. They compared plasmatic anticoagulated patients with patients who underwent antiplatelet therapy and found the last mentioned to have fewer bleeding complications and shorter hospital stays than their counterparts [5]. Leyh-bannurah et al. [6] reported that radical prostatectomy both open and robotic-assisted can be safely performed  under continued aspirin [6]. Sforza and colleagues found nerve-spared anticoagulated patients to experience more bleeding complications [7]. Kobuta and associates performed RARP under continued anticoagulation and antiplatelet therapy [8]. Nonetheless, the evidence on the impact of anticoagulation drugs on the outcomes of RARP is limited and not conclusive. This study aims to explore the safety of RARP in this cohort in a real-world scenario without any exclusion criteria, with almost 40% locally advanced carcinomas.

## Methods

All procedures (*n* = 500) were concluded with the Da Vinci X® Surgical System (Intuitive Surgical, Sunnyvale, CA, USA). Pelvic lymphadenectomy was done in all cases. The urinary diversion was carried out through a transurethral catheter (TUC), which was removed on the first postoperative day (POD), and a suprapubic (SPC) catheter. On POD3, patients were allowed to urinate naturally. The suprapubic catheter was removed after one day when micturition was successful without post-void residual urine. In cases of primary extravasation on cystography, patients were discharged with catheters, and the catheters were later removed during an outpatient visit.

500 consecutive patients from a prospectively collected database who underwent RARP between April 2019 and August 2022 performed by a specialized surgeon were included in the analysis. *N* = 124 Patients, 24.8%, received diverse anticoagulants (AC) due to preexisting cardiological disease or coagulation disorder. In our department, routinely all patients receive a subcutaneous low molecular heparin (LMWH) injection once daily starting on the day before the surgery for 3 weeks. For patients under vitamin K antagonists (VKA), the antagonist will be paused 10 days pre- and postoperatively and they will receive LMWH with the doses being adapted to the initial indication for which the antagonist was prescribed. Men under aspirin will continue their medication with no interference and they will receive their LMWH injection additionally. New oral anticoagulant (NOAC) patients stopped their anticoagulation medication 1–2 days preoperatively and continued it on the second postoperative day when there was no clinical sign of postoperative bleeding or gross hematuria requiring intervention. In case the underlying cardiological disease or the coagulation disorder indicates a bridging, this will be done through LMWH. For patients on dual-antiplatelet therapy, clopidogrel was paused 1 week preoperatively and was resumed 1 week after the procedure. Those patients also received LMWH up to 3 weeks after surgery. Patients were divided into 2 groups: group 1 with 376 men, 75.2% received no anticoagulation. Group 2 consisted of 124 patients with different forms of anticoagulation grouped (24.8%). The group 2 was divided into 4 subgroups: subgroup 2A: the aspirin subgroup with 78/500 men, 15.6%; group 2B: the new oral anticoagulants (NOAC) subgroup with 27/500 men, 5.4%; group 2C: the Vitamin K antagonist (VKA) subgroup with 10/500 men, 2%, and group 2D: the dual-antiplatelet therapy (DAPT) subgroup with 9/500 men, 1.8%. We compared and analyzed all demographic and perioperative parameters between groups such as age, the American Association of Anesthesiology morbidity score (ASA), prostate volume in the transrectal ultrasound (TRUS), body mass index (BMI), preoperative hemoglobin (Hgb), the international prostate symptom score (IPSS), and the international index of erectile function (IIEF) questionnaire. Postoperative complications were graded using the Clavien–Dindo classification. Complications and readmission rates were noted for the first 90 days postoperatively.

This study’s design is based on a retrospective cohort study. Statistical analysis was performed using SPSS® v27. Categorical variables were summarized as frequencies (percentage) and continuous variables as mean ± standard deviation and median values. The Kolmogorov–Smirnov one-sample test was used to verify normal distribution. Matched-pair analysis using the independent *T*-test for parametric numeric variables and the Mann–Whitney U test for nonparametric variables was performed. Pearson’s Chi-square test was also used to compare relative frequencies. A one-way ANOVA test was performed for parametric numeric variables. A post hoc comparison (Bonferroni) test was performed in case of a significant ANOVA test result. The independent samples Kruskal–Wallis test was performed for nonparametric variables.

The study was conducted in accordance with the ethical standards of the Declaration of Helsinki and approved by the ethics committees of the medical association Westfalen-Lippe and Wilhelm’s University of Münster (2022-585-f-S).

## Results

### Baseline parameters

Group 2 patients were found to be older than those in group 1, with a median age of 71 years compared to 67 years (*p* = 0.030). When comparing subgroups, VKA subgroup patients had the highest median age of 71.5 years, followed by aspirin and NOAC patients with a median age of 71 years (*p* = 0.001). Patients receiving anticoagulation (AC) had more comorbidities, as indicated by a significantly higher ASA score than those in group 1 (*p* = 0.001). Specifically, the 2D (DAPT) group had the highest percentage of patients with an ASA score of 3, at 77.8%, followed by 2C (VKA) and 2B (NOAC) patients with 50% and 33.3%, respectively (*p* = 0.001). The study subgroups also differed in terms of preoperative hemoglobin (Hgb) levels, with the 2C (VKA) subgroup having the lowest median value of 13.3 g/dL (*p* = 0.001). However, all other parameters were similar among the study groups and subgroups. Please refer to Tables 1 and 2 for further details.

**Table 1:** Analysis of demographic and baseline characteristics

	Total		Group 1	Group 2	*p* value
	Cohort (500)		376 (75,2)	124 (24,8)	
Age (year)					
Mean ± SD	66.8		66 + 7.3	69.6 + 5.7	0.03
Median	68		67	71	
BMI (kg/m^2^)					
Mean ± SD	28.4		28.4 + 4.5	28.9 + 3,8	0.492
Median	28		28	28	
ASA score					
1		99 (19,8)	89 (23,6)	10 (8)	0.001
2		317 (63,4)	249 (66,2)	68 (54,8)	
3		84 (16,8)	38 (10,1)	46 (37)	
Preoperative Hgb (g/dl)					
Mean ± SD		14.7	14.8 + 1.1	14.1 + 1.67	0.001
Median		14.8	14.9	14.5	
IPSS					
Mean ± SD		11.4	11.2 + 8.1	12.2 + 8.9	0.356
Median		14.8	10	10	
IIEF					
Mean		15.2	15.7 + 8.4	13.76 + 9.1	0.311
Median		17	17	13	
Initial PSA (ng/ml)					
Mean ± SD		14.8	15.8 + 8	13.4 + 27	0.341
Median		8	8	7.5	
Prostate-volume (ml)					
Mean ± SD	49		49.9		0.565
Median	43		45	48.4 + 28.2	
D’Amico risk classification					
Low risk	117 (23,4)		92 (24,5)	25 (20,2)	0.341
Intermediate risk	229 (45,8)		171 (45,5)	58 (46,8)	
High risk	154 (30,8)		113 (30,1)	41 (33,1)	
NHT	55 (11)		40 (10,6)	15 (12,1)	0.653
Previous TUR-P	34 (6,8)		22 (5,9)	12 (9,7)	0.143
Nerve sparing					
Bilateral	374 (69,4)		267 (71)	80 (64,5)	0.117
Unilateral	134 (26,8)		94 (25)	4 (3,2)	
No	19 (3,8)		15 (4)	40 (32,3)

**Table 2:** Analysis of demographic and baseline characteristics, compared among subgroups

	Total (500)	Group 1	Group 2A	Group 2B	Group 2C	Group 2D	*p* Value
		376 (75.2)	Aspirin	NOACs	Vitamin K antagonist	DAPT	
			78 (15,6)	27 (5,4)	10 (2)	9 (1,8)	
Age (year)							
Mean ± SD	66.8	66	68.7	69.4	71.3	69.2	0.001
Median	68	67	71	71	71.5	70	
BMI (kg/m^2^)							
Mean ± SD	28.4	28.4	28.39	28.33	27.57	27.4	0.640
Median	28	28	28	30	28	28	
ASA score							
1	99 (19,8)	89 (23,6)	7 (9)	3 (11.1)	0	0	0.001
2	317 (63,4)	249 (66,2)	46 (59)	15 (55.6)	5 (50)	2 (22.2)	
3	84 (16,8)	38 (10,1)	25 (32.1)	9 (33.3)	5 (50)	7 (77.8)	
Preoperative Hgb (g/dl)							
Mean ± SD	14.7	14.8	14.1	14.8	13.4	13.3	0.001
Median	14.8	14.9	14.3	14.9	13.3	14	
IPSS							
Mean ± SD	11.4	11.1	11.8	12.7	13.5	12.5	0.828
Median	14.8	10	10	10.5	12	13	
IIEF							
Mean	15.2	15.7	14.3	12	13.29	13.6	0.309
Median	17	17	13	9	14	16	
Initial PSA (ng/ml)							
Mean ± SD	14.8	15.8	12.2	13.9	9.4	6.2	0.264
Median	8	8	7.6	9	6.1	5.7	
Prostate-volume (ml)							
Mean ± SD	49	49.9	47.8	57.8	43.8	40	0
Median	43	45	42	46	39	43	
D’amico risk classification							
Low risk	117 (23,4)	92 (24,5)	16 (20,5)	6 (22,2)	1 (10)	2 (22,2)	0.944
Intermediate risk	229 (45,8)	171 (45,5)	38 (48,7)	12 (44,4)	4 (40)	4 (44,4)	
High risk	154 (30,8)	113 (30,1)	24 (30,8)	9 (33,3)	5 (50)	3 (33,3)	
Nerve sparing							
Bilateral	374 (69,4)	267 (71)	26 (33.3)	10 (37)	3 (30)	1 (11.1)	0.51
Unilateral	134 (26,8)	94 (25)	48 (61.5)	17 (63)	7 (70)	8 (88.9)	
No	19 (3,8)	15 (4)	4 (5.1)	0	0	0	

Categorical data are presented as numbers %, *NOAC*s novel oral anticoagulant drugs, *DAPT* dual-antiplatelet therapy, *BMI* body mass index, *ASA* American association of anesthesiology morbidity score, *Hgb* hemoglobin, *IPSS* International prostate symptom score, *IIEF* International index of erectile function, *PSA* prostate-specific antigen

### Intraoperative data

Pathological results were similar in all study groups and subgroups. Overall, the median difference in Hgb value before and after surgery was 2.6 g/dl, with no statistical difference found between Study groups and subgroups (*p* = 0.152 and *p* = 0.592, respectively). Despite a trend for higher lymph node involvement in 2B (NOAC) patients (25.9%), the statistical analysis resulted in no significant difference in this regard (*p* = 0.138). Positive surgical margins were generally low, with a total of 7.2%, and were similarly distributed among groups and subgroups. Suprapubic catheter days were longer in group 2 (4.5 vs 4 days, *p* = 0.001). The longest catheter days were observed in the 2B group (NOAC patients) (14 days), followed by 2D (DAPT) and 2C (VAK) patients, with a median of 12 and 9 days, respectively (*p* = 0.007). Overall, 7 out of 500 patients (1.2%) received blood transfusions, with no statistical difference among study groups and subgroups (*p* = 0.273 and 0.944, respectively). For more information, please refer to Tables 3 and 4.

**Table 3:** Intra- and postoperative data and pathological findings between the groups:

	Total (500)	Group 1	Group 2	*p* value
		376 (75.2)	124 (24,8)	
Console time (minute)				
Mean ± SD	151 + 45	152 + 40	148 + 44	0.971
Median	140	140	137	
Prostate weight (g)				
Mean ± SD	61 + 25.6	62 + 25	60.7 + 24.8	0.156
Median	55	56.5	54	
Pathological stage				
pT0	1 (0,2)	1 (0,3)	0	0.095
pT1	1 (0,2)	1 (0,3)	0	
pT2	295 (59)	226 (60,1)	69 (55,6)	
pT3	183 (36,6)	131 (34,8)	52 (41,9)	
pT4	20 (4,0)	17 (4,5)	3 (2,4)	
Positive surgical margins (total)				
< 3 mm	18 (3,6)	10 (2,7)	8 (6,5)	0.219
> 3 mm	18 (3,6)	14 (3,7)	4 (3,2)	
Number of lymph nodes				
Mean ± SD	19.6 ± 7.4	19.64 + 6.9	19.8 + 8.7	0.906
Median	18	18	18	
Lymph nodes metastasis	89 (17,8)	60 (16)	27 (21,8)	0.139
Difference in pre- and postoperative HGB (g/dl)				
Mean ± SD	2.5 ± 4.8	2.72 + 1.29	2.66 + 1.5	0.152
Median	2.6	2.7	2.4	
Length of hospitalization (days)				
Mean ± SD	5.6 ± 1.5	5.52 + 1.7	5.7 + 1.4	0.292
Median	5	5	5	
Catheter days				
Mean ± SD	6.9 ± 4.7	6.6 + 4.5	7.48 + 5.6	0.001
Median	5	4	4.5	
Catheter removed before discharge	368 (73.6)	281 (74,7)	87 870,2)	0.317
Transfusion	7 (1,2)	4 (1.2)	3 (2,4)	0.273

**Table 4:** Intra- and postoperative data and pathological findings among subgroups:

	Total (500)	Group 1	Group 2A	Group 2B	Group 2C	Group 2D	*p* value
		376 (75.2)	Aspirin	NOACs	Vitamin K antagonist	DAPT	
			78 (15,6)	27 (5,4)	10 (2)	9 (1,8)	
Console time (minute)							
Mean ± SD	151 + 45	152	150	150	137	132	0.633
Median	140	140	140	135	130	120	
Prostate weight (g)							
Mean ± SD	61 + 25.6	62 + 25	60.8 + 22	65 + 33.5	58.6+ 19.7	49.6 + 14.7	0.597
Median	55	65.5	54	56	58	51	
Pathological stage							
pT0	1 (0,2)	1 (0,3)	0	0	0	0	0.767
pT1	1 (0,2)	1 (0,3)	40 (51,2)	0	0	0	
pT2	295 (59)	226 (60,1)	35 (44,8)	15 (55,5)	7 (70)	7 (77,8)	
pT3	183 (36,6)	131 (34,8)	3 (3,8)	12 (44,5)	3 (30)	2 (22,2)	
pT4	20 (4,0)	17 (4,5)	0	0	0	0	
Positive surgical margins (total)							
< 3 mm	18 (3,6)	10 (2,7)	6 (7,7)	2 (7,4)	0	0	0.218
> 3 mm	18 (3,6)	14 (3,7)	3 (3,8)	0	0	1 (11,1)	
Number of lymph nodes							
Mean ± SD	19.6 ± 7.4	19.74 + 6.9	18.5 + 7.9	17.67 + 9.8	20.9 + 8.3	20 + 8.3	0.891
Median	18	18	17	12	20.5	20	
Lymph nodes metastasis	89 (17,8)	60 (16)	18 (23,1)	7 (25,9)	1 (10)	1 (11,1)	0.138
Difference in pre- and postoperative Hgh (g/dl)							
Mean ± SD	2.5 ± 4.8	2.56	2.7	3.7	2.55	1.65	0.592
Median	2.6	2.4	2.6	3	2.55	1.65	
Length of hospitalization (days)							
Mean ± SD	5.6 ± 1.5	4.9	5.64	5.33	5	5	0.228
Median	5	5	5	5	5	5	
Catheter days							
Mean ± SD	6.9 ± 4.7	7.2	4.7	10.7	9	12	0.007
Median	5	4	4	14	9	12	
Catheter removed before discharge	368 (73.6)	281 (74,7)	57 (73,1)	18 (66,7)	4 (40)	8 (88,9)	0.316
Transfusion	7 (1,2)	4 (1.2)	1 (1.3)	1 (3.7)	1 (10)	0	0.944

*NOAC*s novel oral anticoagulant drugs, *DAPT* Dual-antiplatelet therapy,

### Complications

Analysis showed no difference regarding overall, minor and major complications between the study groups *p* = 0.160, 0.100, and 0.915, respectively. Readmissions were relatively low (5.6%) and similar between study groups *p* = 0.635. Despite a trend for higher minor complications in 2D (DAPT) and 2B (NOAC) subgroups (both 22.2%) followed by 2A (aspirin) subgroup 19.3%, statistical analysis showed no difference *p* = 0.457. The same was found for major complications and readmissions among subgroups *p* = 0.915 and 0.0635, respectively. The most common minor complication was acute urinary retention (28/500, 5.6%) followed by urinary tract infection (UTI) 2.2% and secondary anastomosis leakage (VUAL) 2.2%. In our series, we noted 4 thromboembolic events without any fatal consequences. Those patients were discharged uneventfully with NOAC for 3–6 months. In our cohort, three men required a second operation. Two of them were operated on for incisional hernias (one on the median mini-laparotomy site and the other on the lateral assistant port site). Only one patient had to be readmitted after discharge due to signs of late intrabdominal bleeding. An emergency laparotomy was conducted, but no active bleeding was found. The patient was discharged with no further complications. Another case of postoperative bleeding was managed conservatively with transfusion with no need for surgical intervention (Tables 5 and 6).

**Table 5:** Complications and readmissions among study groups:

Complications in detail			Total (*n* = 500)	Group 1 376 (75.2)	Group 2 124 (24,8)	*p value*
Overall complications			95	66	29	*0.160*
	Minor		74 (14,8)	50 (13,3)	24 (19,4)	0.100
Minor	CDI 51 (10,2)	VTE	4 (0,8)	2	2	
		Elevated labor parameter	6 (1,2)	4	2	
		AUR	28 (5,6)	20	8	
		Diverse	13 (2,6)	9	4	
	CD II 23 (4,6)	Secondary VUAL*	11 (2,2)	7 (1,5)	4	
		UTI	11 (2,2)	7	4	
		Hematoma requiring transfusion	1 (0,2)	1 (0,2)	0	
	Major		21 (4,2)	16 (4,3)	5 (4)	0.915
Major	CD IIIa 12 (2,4)	NSTEMI	1 (0,2)	0	1	
		Hiatus hernia symptomatic	1 (0,2)	1	0	
		Lymphocele	10 (2.0)	9	1	
	CD III b 8 (1,6)	UUTO	5 (1.0)	5	0	
		Revision	3 (0,6)	0	3	
	CD VI 1 (0,2)	Rhabdomyolysis	1 (0,2)	1	0	
		Readmissions*	28 (5,6)	20 (5,3)	8 (6,5)	0.635

**Table 6:** Complications and readmissions among study subgroups

Complications	Total (*n* = 500)	Group 1 376 (75.2)	Group 2A Aspirin 78 (15,6)	Group 2B NOACs 27 (5,4)	Group 2C Vitamin K antagonist 10 (2)	Group 2D DAPT 9 (1,8)	*p value*
Overall Complications	95	66	18	7 (25,9)	2 (20)	2 (22,2)	*0.785*
Minor							
Minor	74 (14,8)	50 (13,3)	15 (19,3)	6 (22,2)	1 (10)	2 (22,2)	0.457
Grad I	51 (10,2)	36 (9,6)	7	5 (18,5)	1 (10)	2 (22,2)	
Grad II	23 (4,6)	14 (3,7)	8	1 (3,7)	0	0	
Major							
Major	21 (4,2)	16 (4,3)	3 (3,9)	1 (3,7)	1 (10)	0	0.866
Grad IIIa	12 (2,4)	10 (2,7)	2	0	0	0	
Grad IIIb	8 (1,6)	5 (1,3)	1	1 (3,7)	1 (10)	0	
Grad IV	1 (0,2)	1 (0,3)	0	0	0	0	
Readmissions	28 (5,6)	20 (5,3)	7 (9)	1 (3,7)	0	0	0.550

## Discussion

The main finding of our study is that provided the correct management of diverse anticoagulation regimes, RARP presents a safe therapy option to treat men with localized prostate cancer. Thus, patients taking AC did not show a significantly elevated risk for bleeding, thromboembolic, or cardiac adverse events. They also did not experience a significantly higher number of minor or major complications, nor were they readmitted more frequently. In addition, they did not receive more blood transfusions. We observed a slight increase in catheter days for patients undergoing RARP while on plasmatic anticoagulant (AC) or antiplatelet (AP) therapy, with group 2 patients experiencing longer catheter days (4.5 vs. 4 days in group 1). While reports on the safety and effectiveness of RARP under these medications are becoming more common, our study is the first to examine various anticoagulation regimes in a real-world scenario with a substantial number of patients, including those with locally advanced carcinomas and suprapubic urinary diversion, and with no exclusion criteria.

Leyh-bannurah et al. [6] stated that patients who continued taking aspirin medication during radical prostatectomy experienced no significant increase in blood loss [6]. However, in their cohort of open retropubic (RPE) and RARP, patients who continued their aspirin medication needed more blood transfusions compared to those who did not (21% vs. 8% in RPE series and 1% vs. 0% in RARP, respectively). Similarly, Carneiro and colleagues conducted a meta-analysis with 1481 patients and reported no difference in complications between groups, except for transfusion rates (2.6% vs. 1.6%) [9]. In our series, the transfusion rate was also higher in the all-anticoagulant group 2 (2.4% vs. 1.2%) but without any statistical difference (*p* = 0.273). Similarly, Kubota and associates reported no statistical difference in transfusion, interventions, or readmissions in their series of patients who continued their anticoagulants [8]. Krane and colleagues conducted a study to compare patients receiving VKA with controls in two different regimes [4]. In the first regime, VKA was paused a week before surgery and resumed when the transurethral catheter was removed (day 4–21). In the second regime, patients were bridged with LMWH. The study found that VKA patients had increased operative time and longer hospital stays. However, when they compared patients who bridged with those who did not, they found that bridging with LMWH in VKA patients undergoing RARP might increase transfusion rates significantly (23% vs 2%, *P* = 0.042). In our study of patients on VKA, only one patient out of 10 (10%) received a transfusion, but our results must be interpreted cautiously due to the small sample size (*p* = 0.944).

With a study design comparable to our current research, Sforza et al. [7] found that complications and readmissions within 90 days post-surgery did not vary between patients receiving AC and those who did not [7]. However, nerve-sparing procedures resulted in a higher rate of complications and transfusions for patients. In our series, 69.4% of patients received bilateral nerve-sparing, 26.8% received unilateral nerve-sparing and 3.8% did not receive nerve-sparing at all. Given that patients without nerve-sparing were uncommon in our study, we cannot make a similar statement. The authors typically report the amount of blood lost during surgery and have found that patients undergoing anticoagulation therapy experience greater blood loss. [4, 6, 8]. We chose not to use an inaccurate parameter that could be influenced by diuresis or the irrigation method used during the operation. Instead, we measured the difference in hemoglobin (HGB) levels before and after the procedure and found no significant difference among any of the groups or subgroups in our study. Binhas et al., [10] despite relatively higher bleeding events, 33.3% in aspirin-treated patients and 32.5% in controls also found no difference regarding complications postoperatively [10].

In their systematic review including 2516 patients, Ning et al. reported higher bleeding complications and longer hospital stay for anticoagulant patients compared to their antiplatelet counterparts [5]. While Oshima and associates did not find antiplatelet agents to increase bleeding complications when compared to controls, they found anticoagulation agents both VKA and NOAC to increase such complications (4.3% in the AP and 23.5% in the AC group vs. 3.7% in the control group). Interestingly, no significant difference in bleeding complications was observed when the AP was continued or interrupted. Likewise, no difference was to be seen in the heparin bridging and the AC interruption group. Moreover, all bleeding complications observed in the AC group occurred after resuming AC therapy [11]. In our study, median hospital stay days were consistent among study subgroups (median 5 days). Furthermore, no difference in the incidence of complications was noticed when comparing study subgroups (*p* = 0.785). Tamhankar et al. [12] conducted a study comparing patients undergoing RARP under continued 75 mg aspirin and those who did not. Similar to our findings they did not find any difference regarding bleeding complications, yet they found control patients to have a higher incidence of positive surgical margins [12]. In our analysis, the positive surgical margins rate was 7.2%, and was similarly distributed among the study subgroups (*p* = 0.218).

The strength of the study is that it conducted a detailed analysis of pre- and postoperative parameters and outcomes for a large number of patients. The main limitation is that the study was conducted retrospectively. In an attempt to mirror real-world scenarios, the study did not exclude any patients, which meant that results had to be compared between different, inhomogeneous groups of patients undergoing different kinds of anticoagulants and antiplatelets. Therefore, the results should be interpreted with caution due to the small sample size and the retrospective nature of the study. In addition, the study was limited to a tertiary center, so the results may not apply to other centers with lesser operative volumes around the world.

## Conclusion

Under careful management, robot-assisted radical prostatectomy (RARP) is a safe procedure for the population of men with diverse anticoagulation regimes and comorbidities. The functional and oncological outcomes are comparable to those of men with no medication. However, further prospective studies are required to validate our findings.

## Data Availability

No datasets were generated or analysed during the current study.

## Abbreviations

AC: Anticoagulation
ASA: American Association of Anesthesiology Score
BMI: Body mass index
NOAC: New oral anticoagulation
DAPT: Dual-antiplatelet therapy
VKA: Vitamin K antagonist
LMWH: Low molecular weight heparin
Hgb: Hemoglobin
RARP: Robot-assisted radical prostatectomy
IPSS: International prostate symptom score
IIEF: Internal index of erectile function
PSA: Prostate-specific antigen
VTE: Venous thrombotic event
AUR: Acute urinary retention
VUA: Vesicoureteral anastomosis
VUAL: Vesicoureteral anastomosis leakage
UTI: Urinary tract infection
UUTO: Upper urinary tract obstruction
NSTEMI: Non-ST-elevating myocardial infraction
PSM: Positive surgical margins

## References

[1] Sung H, Ferlay J, Siegel RL, Laversanne M, Soerjomataram I, Jemal A et al (2021) Global cancer statistics 2020: GLOBOCAN estimates of incidence and mortality worldwide for 36 cancers in 185 countries. CA Cancer J Clin 71(3):209–249 33538338 10.3322/caac.21660

[2] Ng CT, Bonilla HMG, Bryce AH, Singh P, Herrmann J (2023) Approaches to prevent and manage cardiovascular disease in patients receiving therapy for prostate cancer. Curr Cardiol Rep 25(8):889–899 37490155 10.1007/s11886-023-01909-3 PMC10894683

[3] Davey P, Alexandrou K (2022) Assessment and mitigation of cardiovascular risk for prostate cancer patients: a review of the evidence. Int J Clin Pract 2022:2976811 35685515 10.1155/2022/2976811 PMC9158798

[4] Krane LS, Laungani R, Satyanarayana R, Kaul S, Bhandari M, Peabody JO et al (2008) Robotic-assisted radical prostatectomy in patients receiving chronic anticoagulation therapy: role of perioperative bridging. Urology 72(6):1351–1355 19041033 10.1016/j.urology.2008.06.057

[5] Ning YJ, Wan ZX, Meng J, Wang XP (2022) A systemic review and meta-analysis of the effects of perioperative anticoagulant and antiplatelet therapy on bleeding complications in robot-assisted prostatectomy. Eur Rev Med Pharmacol Sci 26(6):2085–2097 35363358 10.26355/eurrev_202203_28356

[6] Leyh-Bannurah SR, Hansen J, Isbarn H, Steuber T, Tennstedt P, Michl U et al (2014) Open and robot-assisted radical retropubic prostatectomy in men receiving ongoing low-dose aspirin medication: revisiting an old paradigm? BJU Int 114(3):396–403 24127902 10.1111/bju.12504

[7] Sforza S, Grosso AA, Di Maida F, Viola L, Tuccio A, Mari A et al (2022) A comparative study of anticoagulant/antiplatelet therapy among men undergoing robot-assisted radical prostatectomy: a prospective single institution study. J Robot Surg 16(4):849–857 34546522 10.1007/s11701-021-01308-2

[8] Kubota M, Matsuoka T, Yamasaki T, Kokubun H, Hagimoto H, Murata S et al (2021) Effect of continued perioperative anticoagulant therapy on bleeding outcomes following robot-assisted radical prostatectomy. Urology 148:151–158 33248139 10.1016/j.urology.2020.08.095

[9] Carneiro A, Cha JD, Baccaglini W, Husain FZ, Wroclawski ML, Nunes-Silva I et al (2019) Should aspirin be suspended prior to robot-assisted radical prostatectomy? A systematic review and meta-analysis. Ther Adv Urol 11:1756287218816595 30671139 10.1177/1756287218816595 PMC6329037

[10] Binhas M, Salomon L, Roudot-Thoraval F, Armand C, Plaud B, Marty J (2012) Radical prostatectomy with robot-assisted radical prostatectomy and laparoscopic radical prostatectomy under low-dose aspirin does not significantly increase blood loss. Urology 79(3):591–595 22386405 10.1016/j.urology.2011.11.031

[11] Oshima M, Washino S, Nakamura Y, Konishi T, Saito K, Arai Y et al (2021) Risks and complications of robot-assisted radical prostatectomy (RARP) in patients receiving antiplatelet and/or anticoagulant therapy: a retrospective cohort study in a single institute. J Robot Surg 15(4):661–670 33044699 10.1007/s11701-020-01154-8 PMC8295093

[12] Tamhankar AS, Patil SR, Ahluwalia P, Gautam G (2018) Does continuation of low-dose aspirin during robot-assisted radical prostatectomy compromise surgical outcomes? J Endourol 32(9):852–858 29984591 10.1089/end.2018.0390

